# Factors That Control the Force Needed to Unfold a Membrane Protein in Silico Depend on the Mode of Denaturation

**DOI:** 10.3390/ijms24032654

**Published:** 2023-01-31

**Authors:** Nabil F. Faruk, Xiangda Peng, Tobin R. Sosnick

**Affiliations:** Department of Biochemistry & Molecular Biology, Institute for Biophysical Dynamics, The University of Chicago, Chicago, IL 60637, USA

**Keywords:** AFM, magnetic tweezers, simulation, unfolded, denatured, SMFS

## Abstract

Single-molecule force spectroscopy methods, such as AFM and magnetic tweezers, have proved extremely beneficial in elucidating folding pathways for soluble and membrane proteins. To identify factors that determine the force rupture levels in force-induced membrane protein unfolding, we applied our near-atomic-level *Upside* molecular dynamics package to study the vertical and lateral pulling of bacteriorhodopsin (bR) and GlpG, respectively. With our algorithm, we were able to selectively alter the magnitudes of individual interaction terms and identify that, for vertical pulling, hydrogen bond strength had the strongest effect, whereas other non-bonded protein and membrane–protein interactions had only moderate influences, except for the extraction of the last helix where the membrane–protein interactions had a stronger influence. The up–down topology of the transmembrane helices caused helices to be pulled out as pairs. The rate-limiting rupture event often was the loss of H-bonds and the ejection of the first helix, which then propagated tension to the second helix, which rapidly exited the bilayer. The pulling of the charged linkers across the membrane had minimal influence, as did changing the bilayer thickness. For the lateral pulling of GlpG, the rate-limiting rupture corresponded to the separation of the helices within the membrane, with the H-bonds generally being broken only afterward. Beyond providing a detailed picture of the rupture events, our study emphasizes that the pulling mode greatly affects the factors that determine the forces needed to unfold a membrane protein.

## 1. Introduction

In the study of any biochemical reaction, the identification of the rate-limiting step (RLS) is central to the determination of the reaction mechanism. For single-domain soluble proteins, the folding transition state often attains the majority of the protein’s fold, e.g., as defined by contact order [1]. This implies that the search for a conformational ensemble having a native-like topology is rate-limiting [2,3,4]. For error-free tertiary RNA folding, the RLS can be the formation of divalent metal-ion-binding sites [5,6].

However, for membrane protein folding, there have been fewer folding studies examining the rate-limiting events [7,8,9,10,11]. The situation is more complicated because membrane proteins come in two distinct classes, β-barrels and helical proteins, and the folding venue can vary significantly. For example, the native state can be in micelles, bicelles, or bilayers, while the unfolded state can be denatured in solution using denaturants [10], as often performed with soluble proteins, or in a solubilizing detergent such as SDS (which promotes isolated helical structure) [8,9], or the denatured state can reside in a bilayer [12]. Furthermore, the mode of denaturation can vary from heat- or chemical-induced to steric trapping [12], as well as atomic force microscopy (AFM) pulling vertically at one terminus or magnetic tweezers (MTs) pulling laterally (parallel) to the bilayer at two points. The latter two modes have been employed in folding studies of bacteria rhodopsin (bR) [13,14] and rhomboid protease GlpG [15,16], respectively.

Folding simulations have the advantage of being able to selectively alter the various interaction terms, something which would be extremely difficult, if not impossible, to perform experimentally. Our group previously developed force spectroscopy capability for our near-atomic-level *Upside* molecular dynamics (MD) package [17]. The efficiency of our model allowed for pulling simulations on bR with an O(3) slower pulling rate compared to a prior all-atom MD study [18]. Even with the slower pulling rate, *Upside* could complete each replicate within two days’ time on a single core. Although this speed was O(3)–O(4) faster than the experiment, we still were able to conduct sufficient sampling to observe worm-like chain (WLC) behavior, unlike the all-atom study. Our simulations also predicted many of the same intermediates and the reversible folding of helical turns as observed in Perkins et al.’s high-resolution AFM measurement [13].

Our prior study also simulated the lateral pulling of GlpG membrane protein [17] to match the orientation used in an experimental MT study [16]. Different unfolding pathways and intermediates were quantified, and unfolding could begin at either the middle or the N- or C-termini. Both the intermediates observed in the MT experiment were present in the simulation results.

However, in our GlpG simulations, we observed a much larger repertoire of intermediates than observed experimentally [17]. This apparent discrepancy was a consequence of our use of a stiff, AFM-level spring in the initial set of simulations, whereas the effective spring constant was near-zero for MT measurements as the magnetic field varied slowly compared to changes in protein contour length. As a result, the magnetic bead remained in essentially the same magnetic field before and after unfolding; hence, the force remained constant after each rupture event, which is the signature of a very weak spring. Without the relaxation of force after a rupture event, the remaining regions unfolded quicker and more cooperatively in the MT measurements. This difference highlighted how the mode of force application can significantly alter the folding landscape, which should be appreciated when interpreting unfolding data and characterizing rupture events.

Here, we conduct an in-depth study to identify the determinants of the rupture force level observed in force–extension curves (FECs) for bR and GlpG. Our *Upside* MD package is an ideal tool to investigate this question because of its speed and the ability to systematically alter individual energy terms, including H-bond energies and other protein–protein and membrane–protein interactions to probe how the different terms alter the FEC levels. In addition, we introduce an improved protein–membrane energy function. For bR, we also investigate the impact of linker–membrane interactions, considering that charged residues on the linkers must be pulled through the membrane, and we examine the roles of membrane thickness and lateral pressure. We conclude with a discussion of the differences in the dominant interactions defining the rupture forces between bR and GlpG and how these arise from the difference in pulling directions.

## 2. Results

### 2.1. Membrane Burial Potential

Since our 2018 AFM study [17], *Upside*’s forcefield has improved protein–protein interaction terms, which produces better agreement with hydrogen–deuterium exchange data for soluble proteins [19]. Part of this improvement is a consequence of requiring that the simulated unfolded state be expanded with little residual structure. This extra requirement enhances the folding cooperativity.

The major protein–membrane interaction terms are largely determined by the side chain exposure level to the bilayer, the distance from the membrane surface, and H-bonding (Methods). The protein–protein terms have two components: a pairwise interaction and a multibody burial (desolvation) term (as described in earlier work [19,20]). We allowed helices to unfold and refold in the bilayer and solution, which we found to be essential for reproducing realistic trajectories and identifying the factors that determined the rupture forces.

In the present study, we introduce an improved membrane burial potential for our implicit bilayer. Our original membrane burial potential, which also depended on depth, lipid exposure levels, and H-bonding, was formulated based on statistics from the Protein Data Bank (PDB) [21] according to Energy(z,exposure)∝ln(P(z,exposure)) (i.e., inverse Boltzmann). Moreover, it was calibrated separately from the protein–protein terms. In our new version, the membrane potential is trained to replicate the structure of a set of 45 membrane proteins using the same contrastive divergence machine-learning methodology employed for the protein-only terms (Methods) [19]. This two-step training procedure (soluble protein parameters and then membrane protein parameters) results in a better balance of energies. This balancing is especially important for membrane protein unfolding as both the protein–protein and membrane–protein interactions are the dominant factors.

### 2.2. bR Unfolding

**Figure 1:**
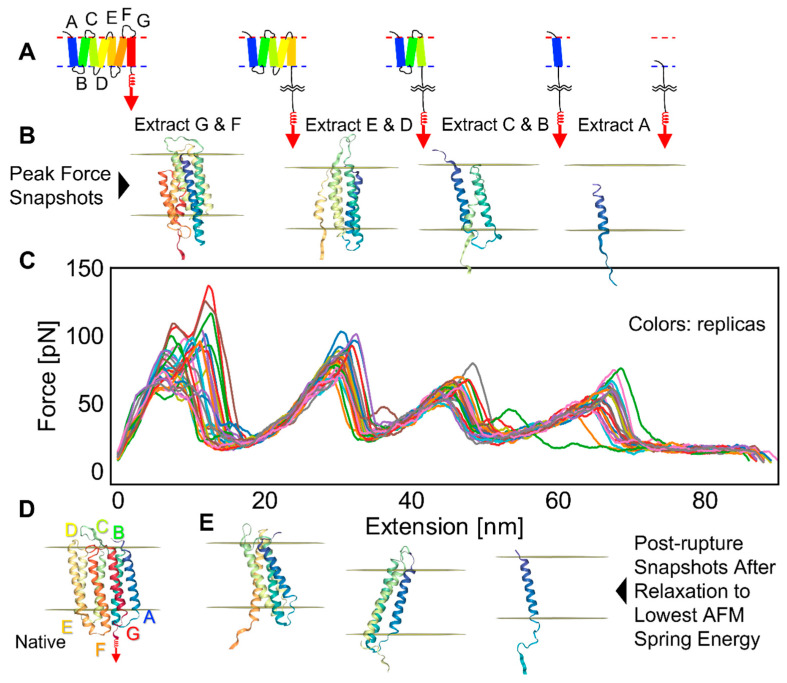
Vertical AFM pulling of bR: (**A**) protein topology and main stages of pulling; (**B**) snapshots from a replica near a rupture event; (**C**) FECs from 28 replicas; (**D**) native structure of bR; and (**E**) snapshots of structures after each rupture at minimum force after AFM spring relaxation. The noticeable divergence of a few FEC profiles is the signature of different unfolding pathways.

We first present the results for the vertical pulling of bR, a light-driven proton pump [13]. The protein consists of seven transmembrane (TM) helices labeled Helix A–G from the N- to the C-terminus (Figure 1). Although bR is part of a homotrimer, we found the unfolding process to be similar for the monomer and homotrimers [17]. Given this similarity, as well as for simplicity, we chose to conduct simulations on isolated monomers. This simplification could result in a decrease in the importance of protein–protein interactions relative to protein–membrane interactions.

In our simulations, an AFM virtual spring was attached to bR’s C-terminus, and the protein was pulled at a velocity estimated to be 10^5^–10^6^ nm s^−1^ using a spring constant of k = 21 pN/nm at 300 K. The four main stages in the unfolding and extraction process are depicted in Figure 1A. Our *Upside* model lacked retinal [14] which is known to interact with the G and F helices. Although this compromised the validity of the force calculations involving these two helices, we present their values as their behavior still can be informative.

The FECs for 28 replicates are shown with illustrative snapshots before and after the rupture event (Figure 1B,D,E). The first six helices primarily came out in pairs (GF, ED, and CB) due to their up–down topology [17]. The FECs exhibited the characteristic sawtooth pattern with the force building up as the virtual cantilever was retracted until a rupture event, whereupon a helical pair was pulled out. The force then decreased rapidly as the slack provided by the newly unfolded portion of the chain (contour length) allowed the cantilever to relax back to its neutral position. Helix A comes out by itself as the final step. Its behavior can be considered representative of the removal of a single transmembrane helix.

The second peak in the FECs corresponds to the unfolding of the ED helix pair. The average rupture force for this unfolding event was 100 ± 2 pN. This value is comparable to the experimental value of 94 ± 1 pN [13], as well as to our previous simulation results of 83 ± 2 pN obtained using an earlier version of our membrane burial potential [17].

It was not surprising that our force values were greater than the experimental values because of our O(4) times faster pulling velocity. The faster force ramp reduced the opportunity for conformational sampling to find lower-energy pathways for unfolding. We previously observed the logarithmic dependence expected from the Bell–Evans theory [22] between the rupture force and the pulling velocity. Figure S1 and Table S1 compare our extrapolation of rupture forces to the experimental pulling velocity using three different simulation velocities. For the ED pair, our previous extrapolated rupture force was 19 pN, while the one for the present study was 34 pN, which still is less than the experimental value of 94 pN. Thus, although we had improvement, there is still room for more. The origins of the difference may include inaccuracies in converting *Upside* time units to real time or deficiencies in the forcefield.

### 2.3. Factors That Govern Rupture Force Levels

**Figure 2:**
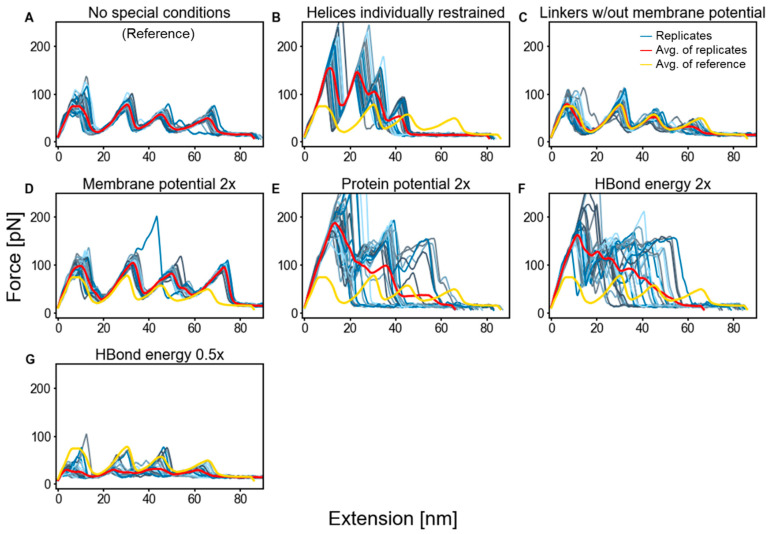
FECs of bR simulations with altered energy terms employing vertical pulling with a stiff spring. Each condition had 28 replicates. The data were smoothed by taking a simple moving average over 50 trajectory frames. In panels (**B**–**G**), the red lines are the average FECs for each of the six altered conditions, while the yellow lines are the average FECs for the unaltered condition from Panel (**A**) and are provided as a reference for the reader’s benefit.

To identify these determinants, we generated dozens of FECs under different conditions (Figure 2) and quantified the rupture forces (Table 1). We started by investigating the effects of constraining the TM helices to remain helical. This constraint had a large effect on the amplitudes of the FECs (Figure 2B), e.g., when the ED helices were pulled out, the average rupture force was 193 ± 6 pN, an increase of 93 pN. The reason for the dramatic increase was that, normally, the C-terminus of Helix E at least partially unfolds prior to extraction. This newly unfolded chain provides enough slack to relieve some or all of the applied tension (Video S1a). Hence, without the possibility of the helices partially unfolding, force levels remained high throughout the extraction process (Video S1b). This result provided insight into the major role that H-bonded structure plays in a rupture event, even if constraining the helices is an unrealistic scenario.

The FECs remained nearly unchanged when the linkers between the helices were excluded from the energy calculation with the bilayer (Figure 2C). Although there were five charged residues distributed among the linkers that were pulled through the membrane (which is unfavorable energetically), turning off the membrane–protein interactions did not produce a measurable change in the rupture force. The ED unfolding event, which involved a Lys in the linker, had an average rupture force of 103 ± 3 pN, which was not significantly different from the original value of 100 ± 2 pN (*p* = 0.427).

To confirm this behavior, we pulled on test systems of single helices with tails that were either neutral (Gly)_5_ or 3e^+^ (Arg)_3_(Gly)_2_. The rupture forces were similar (74.9 ± 1.2 pN and 76.7 ± 1.3 pN, respectively; Figure S2). The lack of a measurable penalty for pulling a 3e^+^-charged linker through the bilayer may be because, once the energy was spent to pull it into the bilayer, the linker required little additional force to be pulled further through the bilayer. This might make the work required for the linker lower in magnitude than for other interactions that must be broken. However, linker charges may affect unfolding pathways as evidenced by hysteresis in equilibrium protein-folding titrations [23], and the noticeable shift in our Figure S2 FECs might reflect this possibility.

We also examined the effect of doubling the strength of different energy terms (Figure 2D–F). The doubling of the protein–protein and H-bonding potentials produced highly variable FECs, which was indicative of increased levels of pathway diversity and, hence, their force levels are omitted from Table 1.

The greatest overall contribution to the rupture force came from the H-bond term. The doubling of this term led to a significant increase in rupture force levels (Figure 2F). The effect of this doubling was similar to that of restraining the TM helices to remain helical, as discussed. Furthermore, the reduction of the H-bond term by 50% noticeably reduced the amplitudes of the FECs (Figure 2G). This reduction at lower H-bond strength indicates that the doubling of H-bonding did not become a rate-limiting factor by overtaking some other factor when it was doubled. This behavior supports our identification of H-bonding being the primary determinant of the rupture levels in this pulling geometry.

For a doubling of the membrane–protein interaction potential, rupture force levels moderately increased (Figure 2D). After excluding the single outlier (large green peak), the ED rupture force was 126 ± 2 pN compared to the original value of 100 ± 2 pN. The outlying FEC was for a pathway where the BC helices were pulled out together with the ED helices in the same rupture event.

As the last helix, Helix A, is the most lipid-exposed at the time of its extraction, it experienced the largest increase in stabilization for a doubling of the membrane burial potential, from 72 ± 2 to 114 ± 1 pN. Table 2 compares the rupture force of Helix A to that of our earlier work and experiment. We previously found that the rupture force was far from the experimental value (23 versus 62 pN) and surmised that the membrane potential could be deficient. The current results (72 ± 2 pN) are improved, which underlines the importance of training the membrane–protein energy term with awareness of the strength of the protein–protein interactions.

**Figure 3:**
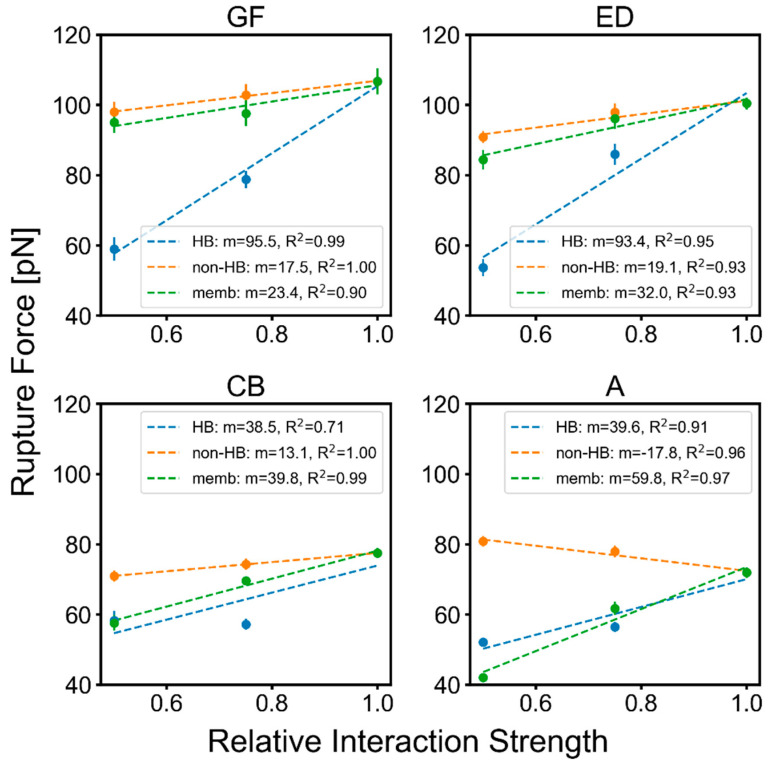
bR helix rupture force trends when scaling different interaction terms. The rupture forces are averages from 28 replicas. HB: H-bonding; non-HB: other protein–protein interactions; memb: membrane potential. The m values are the slope (unit: pN/relative interaction strength).

Generally, the doubling of the strength of the interaction terms increases pathway diversity (e.g., non-overlapping FECs in Figure 2E,F). As this heterogeneity makes quantification of the rupture event difficult, we also examined the effect of decreasing the interaction strengths by factors of ¾ and ½ (Figure 3). For the unfolding of the helical pairs, the rupture forces had a stronger response (steeper slope) to changes in the H-bonding strength compared to changes in the protein–protein or membrane–protein interactions. The GF and ED pairs were only moderately impacted by the membrane–protein potential relative to the other terms.

Although the *Upside* model lacks retinal and, thus, the estimate of the rupture levels for GF were compromised, it is useful to include GF data to illustrate trends for the helical pairs that ruptured later. These later pairs did so with fewer neighbors and, thus, were more lipid-exposed and sensitive to the membrane–protein potential than the helices that unfolded earlier. At the extreme was Helix A, where the rupture force actually experienced a very mild increase with weaker protein–protein interactions. This result is rationalized by Helix A not having many interactions with the other helices prior to unfolding but gaining some low level of non-native interactions during unfolding, with the net result being slight stabilization.

**Figure 4:**
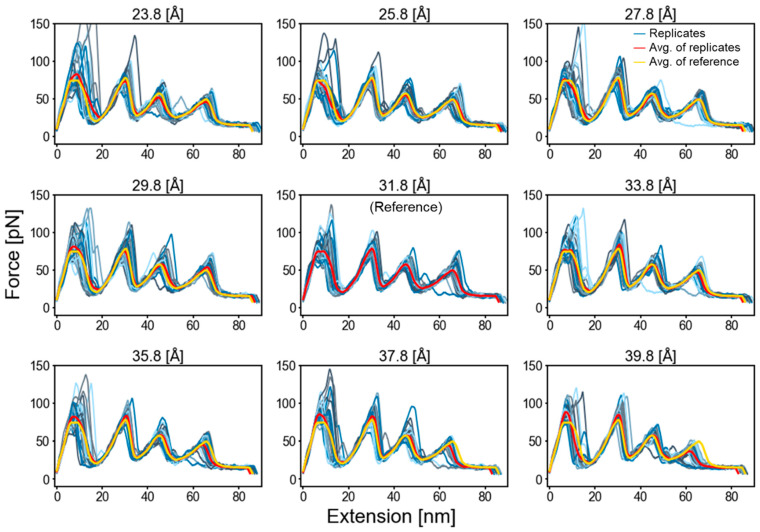
Effect of membrane thickness on 28 FEC profiles. The optimal (lowest energy, center plot) thickness is 31.8 Å [24].

A change in the membrane thickness by up to ±8 Å from the predicted optimal (lowest energy) thickness of 31.8 Å [24] did not have an obvious effect on the rupture force levels (Figure 4). Potentially, the regions of the protein that initially are in contact with the polar lipid head group on the opposite side of the membrane have already been pulled inside the bilayer. Once these regions are no longer in contact with the opposite side’s surface and were only contacting the lipid’s acyl chains, the membrane thickness no longer is a factor.

### 2.4. Characterization of the Rupture Event

**Figure 5:**
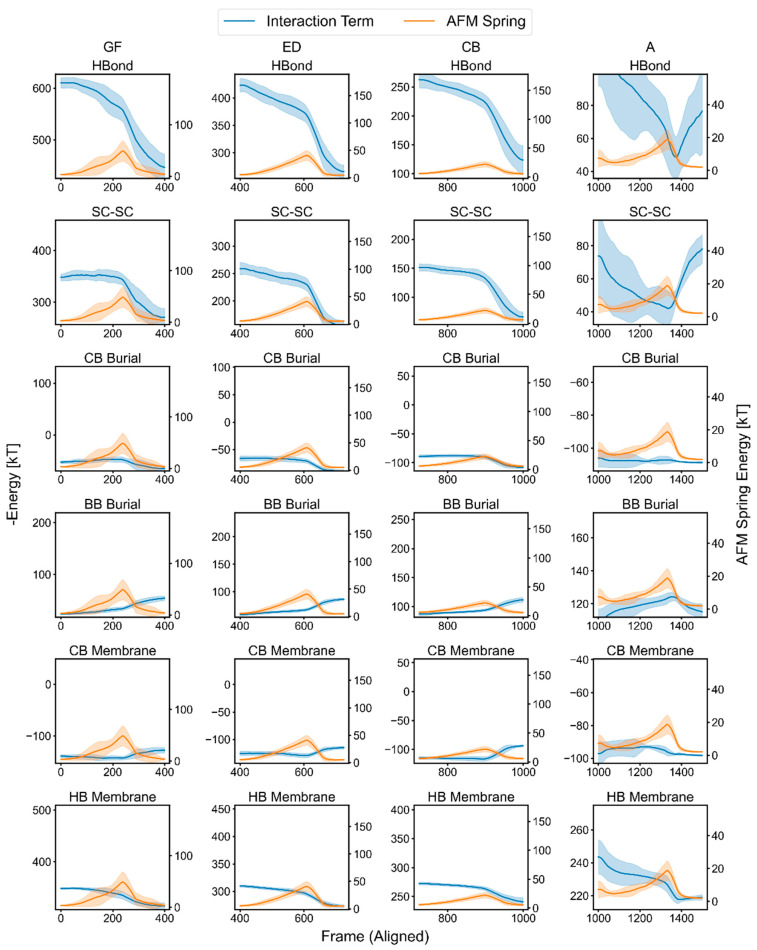
Averaged responses of interaction terms and AFM spring energy during pulling on bR. The blue plots are the negatives of the interaction terms (left y-axis), and the orange plots are the AFM spring energies (right y-axis). The solid lines represent the averages of 28 replicas after smoothing each replica and aligning the maximum spring energy for each rupture event, and the width is the standard deviation. The non-HB terms are further separated into SC–SC (pairwise side-chain interactions) and multibody desolvation terms related to C_β_ and backbone burial. The membrane potential is further separated into CB Membrane (implicit membrane interaction experienced via C_β_) and HB Membrane (perturbation to H bonding in the membrane) (Methods).

The relationship between individual interaction terms and the AFM spring’s potential energy (0.5*kx*^2^, where *k* is the spring constant, and *x* is the displacement from neutral) provides an alternative way to investigate their importance to the rupture event (Figure 5). We used spring energies instead of force because they provided a 1:1 comparison with the interaction term energies. The averages of the interaction and AFM spring energies from the 28 replicas were individually aligned according to their maximum spring energies. We took the negative of the interaction energies because a decrease in value after rupture indicates a loss in those interactions.

We first observed a gradual decrease in H-bond energies as the spring energies increased. This was followed by a pronounced drop in the H-bond energies after the peak of the spring energy (Figure 5, top row). Similarly, there was generally a gradual decrease in pairwise protein–protein side-chain energies (“SC–SC”), although the slope was shallower than those observed for the H-bond energies. After rupture, more interactions were rapidly lost as the helices separated from their neighbors.

As discussed above, Helix A was unique in that it did not have any helix neighbors during its extraction, which was reflected by its protein–protein energy being at a minimum before rupture. H-bond and protein–protein energies actually increased slightly after the rupture of Helix A because the entire protein was free of the membrane anchor, allowing it to relax and form non-native interactions.

We also observed unfolding transitions in the protein–membrane energy, as well as in the Cβ and backbone burial energies, although these changes were smaller in magnitude compared to the H-bond and protein–protein energies. An exception was Helix A, whose change in the membrane potential component that mediated H-bonding (HB Membrane in Figure 5) was sizeable in comparison to the change in the main H-bond energy.

The data thus far indicate that, for each pulling stage, the force on the helix increases until a critical level of interaction is lost, after which the force is high enough and the system was destabilized enough for the rest of the helix, as well as the next one, to be pulled out in rapid succession. When combined with the information in Figure 4, H-bonding appeared to be a major factor for all helices. However, we also noticed a large drop in the protein–protein interactions at the point of rupture for the paired helices, which could indicate that the decoupling and turning of the second helix in a pair might play a role in defining the rupture force, a possibility that is discussed in the next paragraph.

**Figure 6:**
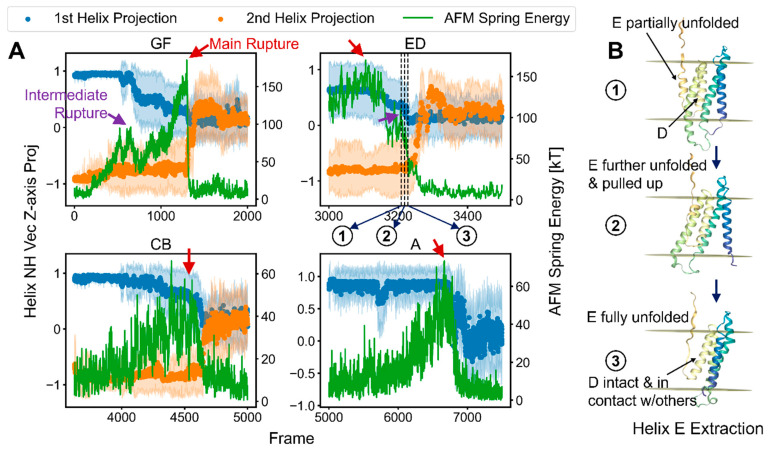
NH bond vector projections onto the z-axis as measures of helix rotation and unfolding. (**A**) Example of projections from a particular replica divided into time frames of the different helix (pair) rupture events. Red arrows point to the main rupture events corresponding to maximum AFM spring energies/pulling forces, while purple arrows provide examples of intermediate rupture peaks that coincided with partial unfolding. (**B**) Trajectory snapshots corresponding to the dotted time points in the ED panel of Subfigure (**A**) highlighting the order of unfolding events of the ED helices.

To examine the relationship between the rupture event and the possible rotation of the second helix, we obtained its orientation from the *z*-axis projection of the average of the NH bond vectors of each helix (which points along the helix axis). We compared the projections to the AFM spring energy. Figure 6A shows an example of this event, where the subfigures focus on the respective time ranges of the different rupture events.

The NH bond vector projections began near the maximum value of +1 for the first helix in the pair (blue points) and −1 for the second helix (orange) because the two helices were pointed in opposite directions. A decreasing pattern of steps for some of the NH bond vector projections was observed for the first helices, which corresponded to their ends gradually unfolding while under tension. The unfolded segments became extended, and their NH vectors were now pointed perpendicular to the membrane plane, which decreased their average *z*-axis projection. The change in the projections for the second helices occurred in a more concerted manner, corresponding to their turning or bending before unfolding. Figure 6B examines trajectory snapshots of the ED helices unfolding (corresponding time points indicated in Figure 6A), where it is revealed that the first helix fully unfolded before the second helix began to turn, but only after the tension had already decreased to near-zero. In this rupture, the second helix’s turning was after the rupture event.

**Figure 7:**
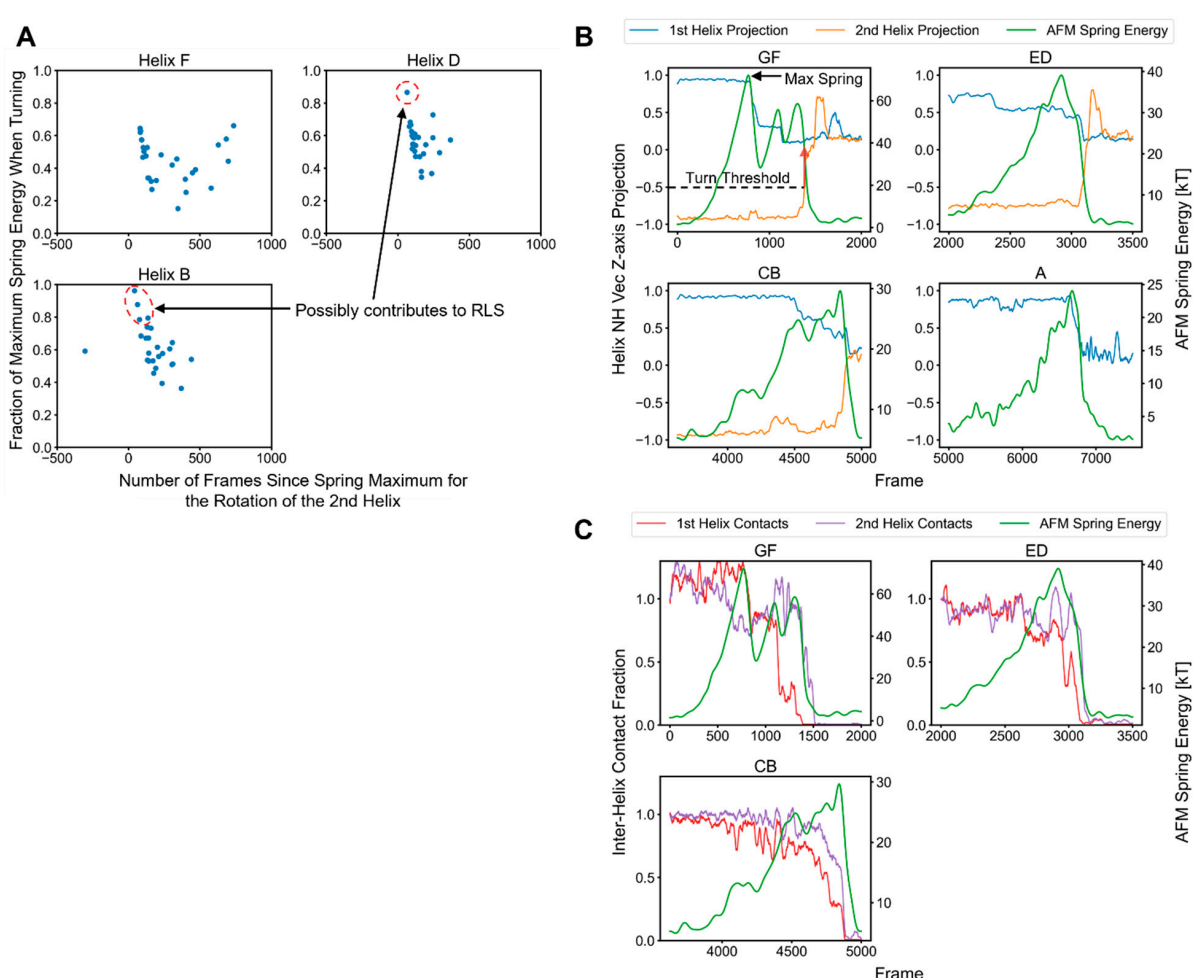
Deconstructing the contributions of non-H-bond interactions to the rupture events. (**A**) The proximity of the second helix’s turning point to the maximum AFM spring energy and the fraction of the spring energy at which this helix turned. Each data point corresponds to one of the 28 replicas. The red circles identify where the turning of the second helix might contribute to the main rupture event (using an 80% cutoff). (**B**) Helix NH bond vector projections onto the z-axis for a particular replica in comparison to the spring energy. The data were smoothed with uniform and Gaussian filters (Methods) unlike those in Figure 6. (**C**) Fractions of the inter-helix contacts of the first and second helices to the other bR helices in relation to the AFM spring energy. These were referenced to the number of contacts at the start time shown for each panel. Helix A is omitted because it lacked a partner at the time of its rupture.

To determine how this helical rotation generalized across the different helix pairs and replicas, we extracted the time points when the second helix began to turn in relation to the rupture event and the fraction of the rupture force at which these occurred (Figure 7A). The threshold for the turning event was taken to be when the second helix NH projection crossed −0.5; at this point, the spring energy was read and divided by the maximum to give the locations and values of the points (arrows and labels in GF panel for Figure 7B). For Helix F, many points for the different replicas occurred long after the peak rupture force (i.e., at x > 250 frames, with x = 0 being the time of the peak force) and at lower forces, indicating that the turning of the second Helix F was usually not part of the rupture event, which was instead defined primarily by a loss in interactions with the C-terminal Helix G.

However, for Helix B and, to a lesser extent, Helix D a few points close to the time and energy of the rupture event indicated that these second helices partially contributed to the force levels in some unfolding pathways (Figure 7A red circles). Considering that the linker between the CB helices was the longest, Helix B’s turning so close to the rupture event implied that turning is mediated by non-bonded interactions. Figure 7B presents the NH bond vector projections in comparison to the spring energy for a particular replica, as in Figure 6, but for a different replica. We saw distinct spring energy peaks for the unfolding of the GF helices, with the second helix turning long after the maximum spring energy level. Hence, for this helical pair, the unfolding of each helix typically was a distinct event, with the rotation of the second helix occurring separately.

The maxima of the spring energies for all the helices did have some degree of correspondence to a response in the NH vectors, particularly those of the first helix (or Helix A), indicating that H-bonding was involved in defining the rupture. Figure 6C contains plots for the same replica but compares the fractions of inter-helix contacts and the spring energy. The fraction of contacts was referenced to the start of the time range for the rupture events and was calculated between the target helix and all the other helices. Here, we can see that the rupture peaks corresponded to the first and, sometimes, the second helices, losing contact with the other helices, particularly for the CB pair. Thus, in combination with the scaling studies, we can conclude that H-bonding was the major contributor to the rupture force, but other non-bonded protein interactions also play a role.

We also investigated the effect of lateral pressure on the extraction of Helix A with an extended linker segment on the C-terminal side attached to the AFM spring (Figure S3). The application of >10^3^ bars of compressive or tensile pressure was required to noticeably observe a change in the rupture force, whereas membrane proteins in resting membranes only experience a few hundred bars of compression and up to about one thousand bars of tension in localized areas from interactions with lipids [25]. Hence, we inferred that lateral pressure is not likely to be a significant factor in the force levels for extracting individual helices from bilayers.

### 2.5. GlpG Lateral Pulling

**Figure 8:**
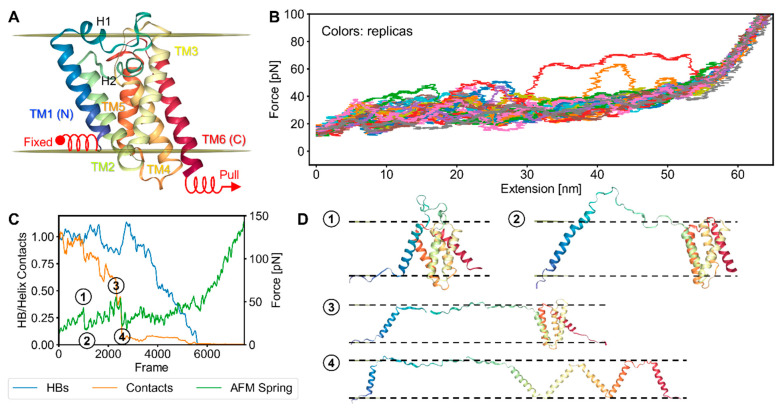
FECs for the unfolding of GlpG upon lateral pulling: (**A**) structure of GlpG and AFM spring attachment points; (**B**) FECs of 28 replicas (displacement of the fixed end is subtracted); (**C**) response of H-bonds and inter-helix contacts to the AFM spring force for a particular replica presented as a fraction of their counts in the starting native structure; (**D**) snapshots corresponding to the numbered rupture and relaxation points in Subfigure (**C**).

We contrasted the bR results with an investigation of rupture events when unfolding occurred via force being applied laterally, as was performed experimentally on GlpG [16]. GlpG is an *Escherichia coli* rhomboid protease with six TM helices (Figure 8A). We used the same spring constant and cantilever velocity as in the bR simulations and pulled from the carboxy terminus. In addition, GlpG’s N-terminus was held in place with a spring, and tension was applied in the plane of the bilayer rather than vertically in the z-direction.

The lateral pulling on GlpG’s termini gave rise to a more heterogeneous set of FECs that generally had lower rupture forces compared to the vertical pulling on bR. The heterogeneity indicates that there was more pathway diversity for GlpG due to tension being applied at both the N- and C-termini, whereas only the C-terminus experienced tension in the bR simulations, and unfolding was a vectorial C-to-N process (Figure 8B). Furthermore, for GlpG, the rupture peaks were not as easily distinguishable or synchronized across replicas as bR’s relatively reproducible sawtooth pattern. As a result, we examined the replicas individually, comparing the fraction of H-bonds and inter-helix contacts to force levels (Figure 8C; see supplementary Video S2 for the trajectory of the shown replica). For GlpG, the drops in inter-helical contacts usually corresponded better with drops in the force compared to drops in the number of H bonds. This difference indicates that non-H-bonding interactions played a bigger role in the rupture events. The rapid increase in the force at the end of the FECs, when all contacts and H-bonds were lost, was simply due to the chain being stretched to the limit of its contour length.

Snapshots near the rupture event demonstrate how unfolding typically started with the separation of 1–5 helices and a loss of inter-helix contacts (Figure 8D). Due to the applied tension, the initial and subsequent separations sometimes were accompanied by the partial or complete unfolding of some of the helices. However, in many cases all six transmembrane helices fully separated, even though their helical structure remained mostly intact, e.g., Snapshot 4. Upon further tension, separated helices could unfold and lie on the surface of the bilayer. Most of the helices only began to unfold after full separation.

**Figure 9:**
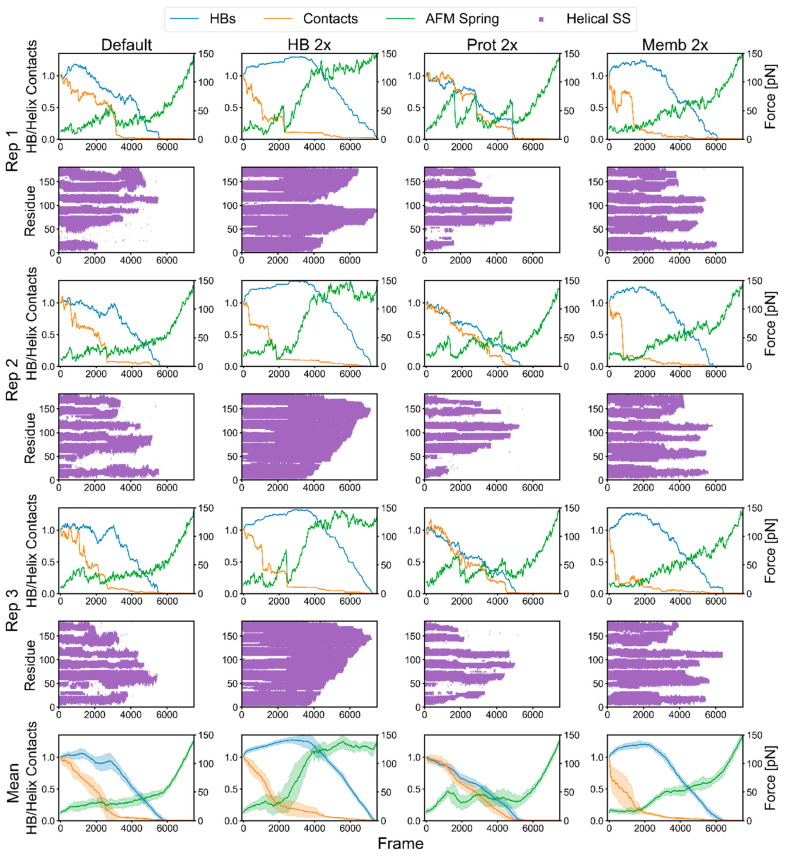
Effects of doubling each of the major energy terms on the lateral pulling of GlpG. The interaction and force plots for each replica are accompanied by a timeline of helical structure below (purple bars). Columns are forcefield conditions and rows are replicas. First column: standard forcefield; second: H-bond energy doubled; third: non-HB potential doubled; fourth: protein–membrane potential doubled. Last row are the average results of 28 replicas.

Figure 9 highlights how the interactions and force varied across replicas (rows) and conditions (columns). Three individual replicas are included for each condition, while the last row contains the averages and standard deviations for the 28 replicas. Each individual replica has a corresponding plot presenting the extent of the helical structure (purple bars). These timelines of helical structure indicate that unfolding could begin from the middle or from either end of GlpG. We again found that the early force peaks coincided with losses in inter-helical contacts, whereas the majority of the H-bonds were usually lost afterward (Figure 9, first column).

This finding was supported by simulations where the H-bond energy was doubled but had minimal impact on rupture force levels (Figure 9, “HB 2x”). The intact helices still separated early, and the protein–protein interactions largely defined the magnitude of the early rupture peaks. H-bonds persisted longer in the helices than originally and required much higher forces before unfolding.

With a doubling of protein–protein potential (“Prot 2x”), the force profiles became more pronounced and regular, adopting a more sawtooth-like pattern, although not as well-defined as the bR FECs. Now, H-bonds decreased along with inter-helical contacts, which, not surprisingly, were more persistent than before. Visual inspection of the trajectories showed that unfolding could occur while some helices remained in contact. Finally, with the doubling of the membrane–protein potential (“Memb 2x”), the inter-helical contacts were lost quicker and at a lower force because the helices were better solvated by the bilayer. The average of the 28 replicas obscured the individual rupture peaks but illustrated the order of events, especially the correspondence between the loss in interactions and the rupture events, which later were followed by the loss of the majority of the helical structure (Figure 9, last row).

## 3. Discussion

*Upside’s* combination of speed, accuracy, detail, and control over individual energy terms enabled us to run hundreds of unfolding trajectories across multiple conditions to uncover the factors that governed the rupture forces for two membrane proteins, bR and GlpG. By examining the FECs and altering the strength of individual energy terms, we found that a rupture event is a complicated, multistep process, sometimes with multiple unfolding pathways and a strong dependence on pulling geometry. Nevertheless, we still could deduce general trends.

The general rupture mechanism for bR, which was pulled vertically with respect to the bilayer, was the successive extraction of pairs of helices starting with the GH helices, which were closest to the cantilever. The pairwise unfolding of helices occurred with the change in interactions as the force on the helix closer to the cantilever built up and began to unfold. The interactions that changed included H-bonding, inter-helical contacts, and protein–lipid interactions. The trend was for the force to increase until the first of the helical pairs was pulled out and unfolded. Tension shifted to the second helix of the pair, which, by virtue of the pulling geometry, rotated and followed the exit route of the first helix. For most of the helix pairs (>80%), the complete 180° rotation of the second helix only occurred after the rupture event [26].

We found that H-bond strength generally had the greatest impact on the rupture force, whereas changes in protein–protein and membrane–protein interactions had mixed effects. The latter term grew in importance as fewer, more lipid-solvated helices remained in the bilayer. For the last isolated helix, Helix A, H-bonding and membrane–protein interactions were similar in magnitude. Other factors that had minimal impact included membrane thickness, electrostatic charge on the linkers, and lateral pressure (within reasonable ranges).

For the lateral pulling of GlpG, unfolding could begin at either end or at the middle of the protein, with groups of helices separating from each other before the majority unfolded. The ease with which the various groups of helices separated was determined by the relative strength of the protein–protein and membrane–protein interactions as separation resulted in the helices being better-solvated by the lipids [12,27]. As a result, a helix with a more favorable insertion energy was more likely to separate earlier from the other helices when under force.

For the early peaks in the FECs, the protein–protein interactions were the dominant factor. However, the tension on the helices that separated early could result in their unfolding before the other helices separated, and this unfolding was affected by the H-bond strength. Overall, the H-bonds had decreased importance in lateral pulling measurements compared to vertical pulling and were attributable to the difference in the pulling geometry.

In our previous study [17], we also studied GlpG unfolding using a soft spring to simulate magnetic tweezer (MT) measurement. With a soft spring, the force built up until the first rupture, after which it remained constant as in a force clamp. The retention of force at this high level typically resulted in the remaining portion of the protein also being pulled apart in the initial rupture event. Hence, the same factors that contributed to the first rupture event with a stiff spring should be the same as those for the sole rupture event observed in MT measurements.

A limitation of our model was the use of an implicit membrane, which could miss some of the molecular details. Lipid molecules may shift position as a protein is extracted from the bilayer. For example, during the vertical pulling of bR, nearby lipids could be pulled up along with the protein. Our model did not capture the work required to deform the membrane in this manner, and thus, we may be underestimating the impact of protein–membrane interactions on rupture forces. The disruption in the membrane also could create openings for water to penetrate the membrane and interact with the TM helices, altering the balance of energies. For example, our energy penalty for unsatisfied H-bonds within the membrane would be incorrect if water was available for H bonding. Nevertheless, the major factors governing vertical pulling (H-bond breakage) and horizontal pulling (separation of the helices) are likely to be unchanged with an explicit bilayer.

The present study makes several predictions for bR folding, including the following: (1) The peak force is invariant to membrane thickness or (2) to charged amino acids in the linker. (3) The use of lipids that increase membrane–protein interactions can increase the rupture force. Likewise, the present study makes a number of predictions for GlpG unfolding: (1) Helix separation can begin at either end, but the N-terminal helix preferentially separates first. (2) Loss of helical structure occurs after separation and can begin at either end or at the middle, but there is a C-terminal bias. (3) Increasing membrane–protein interactions may result in the helices separating quicker, and helix unfolding is further biased to start at the C-terminus.

- Comparison to previous studies.

Membrane protein folding studies can be quite diverse, with each study probing a different region of the energy surface (Table 3). For bR and GlpG, we found that the pulling direction is a key determinant of the nature of the rupture event and the unfolding pathway. Pulling measurements typically start from the native state and end with an extended state that may [16] or may not [13] be reversible.

As Yamada et al. found for the vertical pulling of bR, there was a diversity of sub-pathways along the overall C → N unfolding pathway, as well as different contributions to the rupture force [26]. They estimated SC–bilayer interactions to have a larger effect on force levels compared to H-bonds, which our study found to generally be the larger contributor for vertical pulling. Their study and others [28] also have emphasized the role of interactions with the membrane interface. Note that we also observed a greater role for protein–membrane interactions with fewer remaining helices. Finally, while we included inter-helix interactions, Yamada et al. omitted them in their model but acknowledged that, according to other studies [18,29], these interactions could affect force levels and pathway frequencies.

For some membrane proteins, conditions can be found where a chemically denatured protein can spontaneously insert and fold inside the bilayer [10]. SDS denaturation and steric trapping consider different denatured states compared to the extended denatured states pertinent to force spectroscopy experiments, whether they are on the whole molecule or just the probing of a single helix [14]. A large amount of native secondary structure is present in the SDS states of bR and GlpG [8,9], while the sterically trapped state of GlpG is relatively compact, with some helix unfolding and dissociation to the membrane–water interface [12]. However, these states have similarities with lateral pulling, where we observed GlpG’s helices dissociating before most of the helical secondary structure was lost. One key difference is that SDS [9] and steric trapping [12] experiments indicate that helices close to the amino terminus remain intact in the transition and denatured states. This is in agreement with our MT simulations and our experiment that observed a carboxy-to-amino unfolding bias, and it aligns with the in vivo insertion of protein beginning at the amino terminus. However, our results showed that GlpG unfolding could also occur from the N-terminus, suggesting that force can bias the unfolding pathway.

While vertical pulling on membrane proteins generally has the least correspondence to the other modes of denaturation, there are a variety of biologically relevant processes where vertical forces are highly relevant. These include the insertion of helices during biogenesis and co-translational folding [30,31], as well as the BAM chaperone complex where strands can be singly or pairwise inserted into the bilayers in the folding of outer membrane β-barrel proteins [32,33,34]. In sum, there remains a lot of fascinating work to be conducted.

**Table 3:** Folding studies of bR, GlpG, and other membrane proteins.

Protein (Reference)	Study	Reference State	Comments
bR (present study)	Vertical pulling with stiff spring from carboxy terminus	Extended with some residual helical structure after release from membrane	Pairwise unfolding of helices, H-bond strength affects rupture event
bR [35]	φ analysis conducted in SDS	Helical structure due to the presence of SDS	Stable core, polarized transition with some helices structured
bR [14]	AFM studies of point mutant in helices	Extended under tension	ΔΔG for helix to extend under tension, unlike mutational studies with soluble proteins
GlpG (present study)	Lateral pulling with stiff spring	Extended under tension	Unfolding can begin from middle, amino, or carboxy terminus, but some helices separate before unfolding
GlpG [9]	SDS denaturation from mixed micelles using φ analysis	Helical structure due to SDS	N-terminal nucleus involving helix 1 and 2, but with non-native contact in the loops with 3 C-terminal helices unfolded
GlpG [12]	Steric trapping with streptavidin in bicelles and lipid bilayers	Some helix fraying, expanded compared to native state, but still compact	Unfolding and separation at C-terminus or middle with the unfolded or separated helices potentially at membrane–water interface
GlpG [16]	Magnetic tweezers pulling laterally in bicelles	Extended under tension	Mostly cooperative unfolding from carboxy-to-amino with two observed intermediates, suggestive of pairwise unfolding of helices, transition state close to native state
PagP [36]	Urea denaturation from pure liposomes studied using φ analysis	Chemically denatured	Highly polarized transition state lacking β sheet structure, suggests tilted insertion into membrane, folding pathway and transition state affected by lipid composition
KcsA (helical tetramer) [37]	Folding upon transfer from SDS into liposomes	Partially folded monomers	Partially folded monomers rapidly associate, rate-limiting step is unimolecular, possibly relates to formation of the tetramer’s selectivity for filter and pore helices

## 4. Methods

### 4.1. Structure Preparation

The structures of bR (Protein Data Bank (PDB) ID: 1qhj) and GlpG (PDB ID: 2xov) and their positioning within membranes were obtained from the Positioning of Proteins in Membranes (PPM) 2.0 Web Server [24]. The optimal membrane thicknesses (bR: 31.8 Å; GlpG: 28.6 Å) were taken from the PPM predictions as well.

### 4.2. Upside Simulation Settings

The AFM springs were attached to the Cα of the C-terminal residues of bR and GlpG. GlpG also had a spring attached at the N-terminal to anchor it to its starting position, whereas bR’s N-terminus was unanchored. The spring constant used was κ = 0.05 k_B_T/Å^2^ (∼21 pN/nm at 300 K), and the pulling velocity was 0.001 Å/*Upside* time step ≈ 10^6^ nm/s. The temperature in *Upside’s* reduced units was T = 0.85, which we previously established to be approximately 300 K for soluble systems [19]. The correspondence to physical temperature may be different for membrane proteins due to the implicit treatment of lipid interactions, but a highly accurate temperature was not important for the purposes of the studies in this paper. A multi-timestep Verlet integrator [38], newly implemented in *Upside*, was used with slower varying forces calculated three times less frequently than those for fast ones. The frame interval for both the bR and GlpG simulations was 100 *Upside* time units (*ups*). The duration for bR simulations was 700,000–1,000,000 *ups* depending on the interaction, and 1,000,000 ups for GlpG. These durations were achieved within 36 h allotted wall times for replicas distributed among Intel(R) Xeon(R) CPU E5-2680 v4 single cores on UChicago’s Midway2 computing cluster.

Refer to SI Methods for details on our protein membrane burial potential implementation, training procedure, and lateral pressure algorithm.

### 4.3. Data Processing

FECs for bR and GlpG and interaction response plots of bR (Figure 5) were calculated using every five frames of trajectories, whereas the bR NH bond vector and inter-helix contact plots (Figure 6 and Figure 7) and the GlpG H-bonds and inter-helix contact plots (Figure 8 and Figure 9) used every frame. FECs and interaction term response plots were smoothed with a moving average (i.e., uniform filter) having a window size of 50 data points. The other plots used uniform filtering with a window size of 25 data points for a sharper identification of the rupture event. One exception was for the second bR helices’ turning delay analysis (Figure 7), where the AFM spring energies were smoothed with a Gaussian filter with a 25 data point width because this filter is better at preserving edges than uniform filtering (e.g., large drops after rupture events).

## Figures and Tables

**Table 1:** E and D helices’ average rupture force [pN].

Wang et al. [17]	Experiment [13]	Current Potential	Helices Restrained	Linker–Membrane Turned Off	Membrane–Protein Potential Doubled	H-Bond Energy Halved
82.7 ± 2.4	94.1 ± 1	100.4 ± 1.7	193.4 ± 5.9	103.2 ± 3.0	126.0 ± 1.7	53.7 ± 2.4

**Table 2:** Helix A’s average rupture force [pN].

Wang et al. [17]	Wang Membrane–Protein Potential Doubled [17]	Experiment [13]	Current Potential	Membrane–Protein Potential Doubled
22.6 ± 1.6	38.9 ± 2.2	62 ± 0.6	71.9 ± 1.5	114.1 ± 1.2

## Data Availability

The source code, parameters, and usage examples for *Upside* can be obtained from https://github.com/sosnicklab/upside2-md/, accessed on 1 August 2022. The release that corresponds to this work is tagged “fec_factors”.

## Supplementary Materials

The following supporting information can be downloaded at: https://www.mdpi.com/article/10.3390/ijms24032654/s1. Refs. [39,40,41] cited in Supplementary Materials.
