# Supplementary material for: Factors That Control the Force Needed to Unfold a Membrane Protein in Silico Depend on the Mode of Denaturation
Source: Int J Mol Sci. 2023 Jan 31;24(3):2654. doi: 10.3390/ijms24032654 (PMC9917119; doi:10.3390/ijms24032654)
Supplement: Supplementary file 1 [file ijms-24-02654-s001.zip › ijms-2163330-Supplemental Material.pdf]

Supporting Information

SI Methods

Protein-membrane burial potential implementation.

The protein-membrane potential consists of two separate forcefield sub-terms, one acts on the Cβ while the other acts on the NH and CO backbone groups. Each sub-term contains two states. In the sub-term for Cβ, the two states (two potential curves) are for residues exposed on the outer surface of the protein (blue lines in **Fig. S2 A**) and residues buried inside (orange lines), respectively. The height of the curve changes with z, reflecting the energy change during the membrane penetration process. At the same z depth, the difference between the two curves reflects whether a residue prefers protein or lipid. If the blue curve is lower than the orange curve, the amino acid is more likely to be exposed on the outer surface, and vice versa. In the sub-term for the backbone NH and CO groups on the outer surface, there are also two curves for protected NH or CO (by virtue of hydrogen bonding) and unprotected NH or CO respectively, as shown in **Fig. S4 E,F** (the application of force is on N or C atoms).

To improve the training while reducing the risk of overfitting, we trained using the single lipid bilayer energy curves, but apply these curves to both the inner and the outer lipid layer. This procedure assumes that the potential energy function is symmetrical with respect to the center of the bilayer, although in fact some asymmetry exists as accounted for by our previous statistical potential [17]. The complete energy curve is the combined single lipid layer energy curves and two states (**Fig. S4 A-D**):

$$V_{memb,CB}(z,b) = score_{exposed} \times [score_{lower} V_{exposed}(-z-C) + score_{upper} V_{exposed}(z-C)] + score_{buried} \times [score_{lower} V_{buried}(-z-C) + score_{upper} V_{buried}(z-C)]$$

$$V_{memb,NH/CO}(z) = (1-s_{HB}) \times [score_{lower} V_{free}(-z-C) + score_{upper} V_{free}(z-C)] + s_{HB} \times [score_{lower} V_{hbonded}(-z-C) + score_{upper} V_{hbonded}(z-C)]$$

$$score_{lower} = S(z, 1.0)$$

$$score_{upper} = 1.0 - S(z, 1.0)$$

$$score_{exposed} = S(b-2.5, 1.0)$$

$$score_{buried} = S(2.5-b, 1.0)$$

$$C = 0.5 \times thickness$$

$$S(x, sharpness) = \begin{cases} 0.25 \times (x \cdot sharpness + 2) \times (x \cdot sharpness - 1)^2 & if -1 \le x \cdot sharpness \le 1 \\ 0.0 & if\ x \cdot sharpness > 1 \\ 1.0 & if\ x \cdot sharpness < -1 \end{cases}$$

Where z is the z-position, b is the Cβ burial level contributed by the side chain beads in *Upside* model, s_{HB} is the H-bonding score in *Upside* model. $s_{HB} = 1$ is the value of a perfect H-bond, whereas 0 represents no H-bonding; the functional forms of these terms are described in detail in earlier publications [19,20]. C references the z-position of the probe with respect to the upper or lower bilayer boundary, where moving from outside the bilayer to the center from either boundary corresponds to a positive direction in the transformed coordinates. This outside-in referencing across the bilayer boundaries accommodates different membrane thicknesses and also allows the flexible nature of the spline function to focus on details of the potential at the boundaries where there is greatest physiochemical change as opposed to the more uniform center of the bilayer and a referencing from the center-out. $S(x,\ sharpness)$ is a sigmoid-type function that has a compact shape. $V_{exposed}$, V_{buried} , V_{free} , and $V_{hbonded}$ are cubic spline functions with parameters from ConDiv training (will be introduced in the next section). The parameters of $V_{exposed}$ and V_{buried} depend on the residue type. There are thus (20 residue types) × (2 bilayer splines: $V_{exposed}$, V_{buried}) × (18 spline nodes) parameters for the Cβ term. The parameters of V_{free} , and $V_{hbonded}$ depend on the acceptor (CO) or donor (NH) type. So, there are (2 H-bonding types: CO, NH) × (2 states: free or H-bonded) × (18 spline coefficients) parameters for the H-bonding sites on the outer surface.

In addition to the potential curves mentioned above for describing protein-lipid interactions, we also introduced a third curve for the residue on the inner surface of proteins with a solvent channel traversing the bilayer, e.g., an ion channel. The inner surface residue is exposed to the water, not to the lipid, so it should be treated differently. In our model, this potential curve shares the same shape as the curve on the outer surface residue at outside of the lipid region, but is completely independent in the lipid region, as shown by the green line in **Fig. S6**. This potential energy curve was turned off since the simulations in this work did not involve channel proteins. The parameterization of the potential curve will be introduced in the **Protein-membrane potential training** section and the method of identifying the inner surface residue of the channel protein is discussed in the **Lateral pressure** section.

Protein-membrane potential training

The parameters of the protein-membrane potential, namely the cubic spline coefficients, were trained on a dataset of 45 membrane proteins (including a number of ion channel proteins to train the potential energy curves of the inner surface residues), split into three minibatches of 15 proteins and trained for 14 cycles and 1500 *ups* of simulation per protein per cycle starting from the native structure, using the contrastive divergence (ConDiv) procedure detailed in earlier work [19,20]. The essence of our ConDiv training protocol is for each iteration to generate an ensemble of conformations using the current forcefield parameters with finite time simulations. This ensemble is used to update the parameters (by gradient descent) such that the system keeps closer to the native distribution in the next round of simulations, or more specifically, minimizing the Kullback-Leibler (KL) -divergence between the latest round ensemble and the native ensemble.

Our force field FF 2.0 [19] parameters obtained for soluble protein folding [19] were used as a starting point in our new FF 2.1 intended for membrane and soluble proteins. The soluble protein parameters were obtained using our new dual-target training procedure that simultaneously attempts to match both the native and denatured state ensembles rather than just the native ensemble. The target denatured state ensemble was approximated as an expanded random walk, as found experimentally [39–41]. Without a relevant denatured state ensemble for membrane proteins, ConDiv training for FF 2.1 used only the native ensemble as the sole target. FF 2.1 also corrects a minor software error (the Cα-Cβ bond vector had been tilted by 90°).

Lateral pressure

Because *Upside* method does not have explicit lipids or water molecules, we use a multi-step voxel-based approach to find the outer surface of the protein through which to apply lateral pressure or tension (**Fig. S5**):

Step 1. Voxelate the transmembrane using $(6Å)^3$ voxels.

Step 2. Label all voxels as a protein voxel if they contain at least one protein atom.

Step 3A. Label the voxels adjacent to protein voxels as lipid voxels based on a search starting outside the protein (e.g., starting from a voxel located in a corner of the the simulation box).

Step 3B: Label all protein voxels that are next to lipid voxels as protein *surface* voxels. (**Fig. S5 E**)

Step 3C: Mark all lipid exposed residues as an *inner surface* residue if its associated voxel is not marked as protein *surface* voxels. This labeling is relevant for proteins with interior solvent channels.

Step 4: To increase resolution, divide each protein surface voxel into 8 smaller voxels, which then are re-scored as either lipid or protein voxels.

Step 5. Identify the protein *surface* small voxels.

Step 6. Obtain the direction of the lateral force acting on the Cβ atoms on the outer surface by determining the number of small *surface* voxels on each of the four sides (xz_{top}, xz_{low} , yz_{left}, and yz_{right} (the numbers in the yellow squares in **Panel H**).

Step 7A. Identify the small surface voxels containing Cβ atoms as *surface* Cβ small voxels.

Step 7B. Identify the perpendicular directions of the interfaces for the lipid and the surface Cβ small voxels. These determine the direction of the lateral force, pointing from the lipid voxels to the surface CB small voxels.

Step 7C. Determine the number of *surface* Cβ small voxels (the numbers in the cyan squares in **Panel I**).

Step 8. According to the equations in **Panel J**, calculate the force applied to each Cβ in the four directions. These equations distribute the force that is applied to all surface atoms just to the surface Cβ atoms and ensure that the total lateral force sums to 0 and prevent any unphysical translation or rotation of the protein.

Step 9. To improve the accuracy of the lateral force distribution, incrementally rotate the protein 7 times by up to $\pi/4$ and redo the force calculation.

Step 10. Average the 8 forces to obtain the lateral force, f_{unit}.

The z dependence of the lateral pressure is modeled with spline, *E(z),* and the lateral force is calculated according to

$$f_{lateral} = E(z) \times k \times area_{voxel\_side} \times f_{unit}$$

where $area_{voxel\_side}$ the lateral area of a small voxel (3*thickness/12 Å^2), k is a unit conversion factor to *Upside* unit system. If the unit of the lateral pressure curve is in bar, the value of k is 2.428602e-5.

SI Figures and Tables

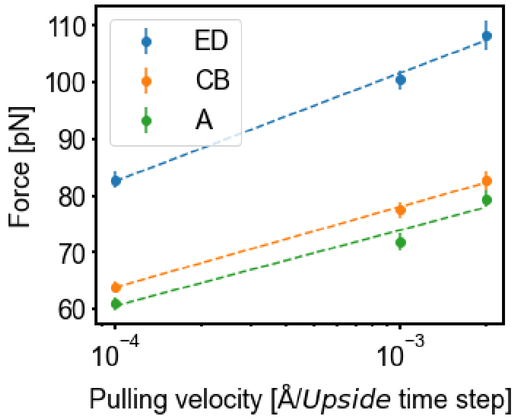

Figure S1. Rupture force scaling fits based on logarithmic dependance on velocity according to the Bell-Evans model.

Table S1. Rupture forces [pN] extrapolated to the experimental pulling speed using the Bell-Evans model fits in Fig. S1. The forces for the simulations were obtained with a pulling speed of 0.001 Å/Upside time units, which we've previously estimated to be 0.001 Å/100 ps. The experimental speed was 300 nm/s = 3 × 10^{-7} Å/100 ps. *Standard errors not reported.

Helices	Previous simulated	Previous extrapolated*	This work simulated	This work extrapolated	Experiment
ED	83 ± 2	19	100 ± 2	34 ± 2	94 ± 1
CB	44 ± 2	9.4	78 ± 1	28 ± 1	49 ± 2
A	23 ± 2	-32	72 ± 2	27 ± 3	62 ± 1

Video S1a: Trajectory of a replica of vertical bR pulling under normal conditions and the pulling force at each frame.

Video S1b: Trajectory of a replica of vertical bR pulling with helices individually restrained.

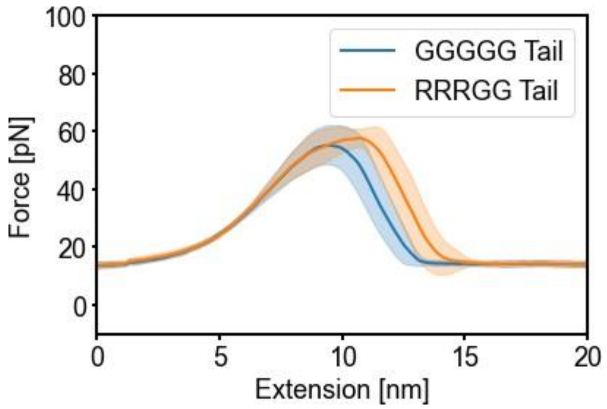

Figure S2. Smoothed FECs from AFM pulling of a single helix with different tail compositions. The legend denotes the tail composition that must be pulled into and through the membrane.

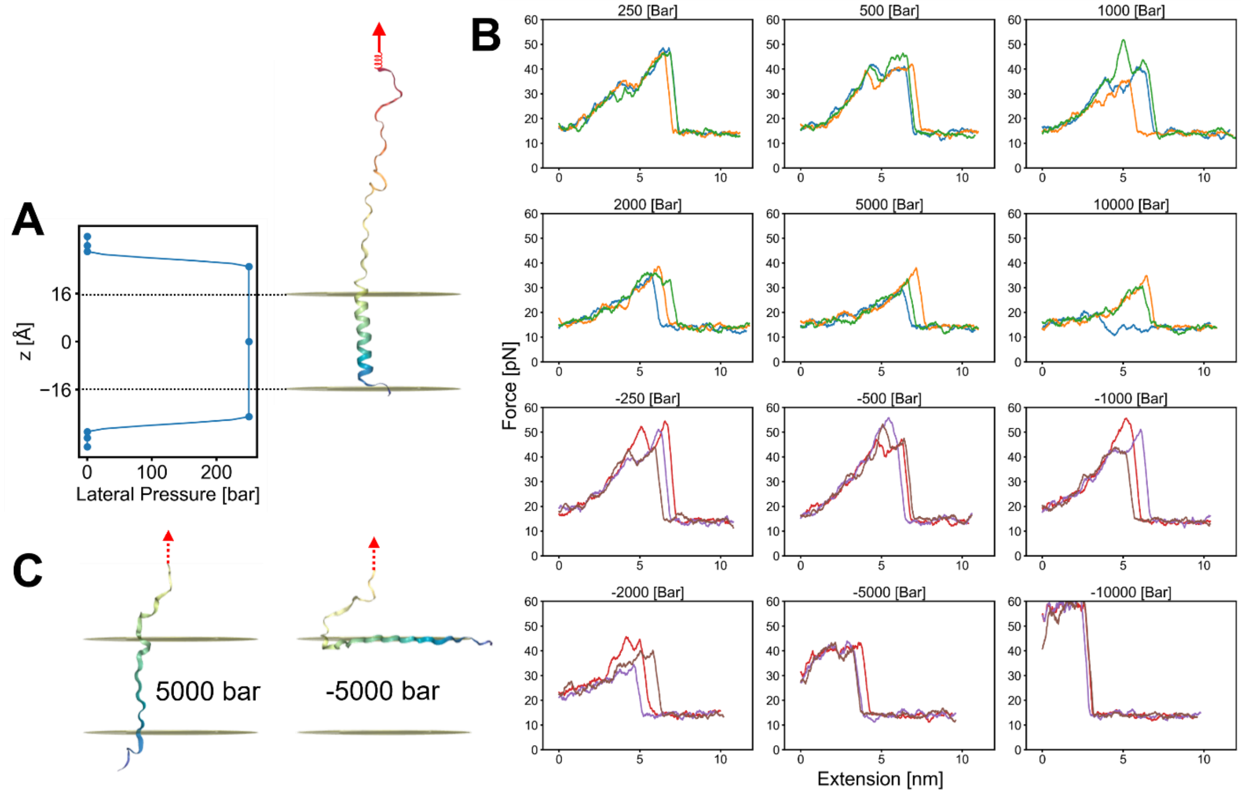

Figure S3. Effect of lateral pressure on FECs of AFM pulling of a single helix. A. Flat lateral pressure profiles were applied (only experienced within the membrane region) to a protein consisting of Helix A from bR plus an unfolded segment acting as a linker. B. FECs from different compressive (top half) and tensile (bottom half) pressures. There are three replicas per pressure. C. Snapshots of typical structures at extreme pressures shortly after the start of simulations.

Video S2: Trajectory of a replica of lateral GlpG pulling under normal conditions. In this example, the protein loses helical secondary structure only after the helices have fully separated.

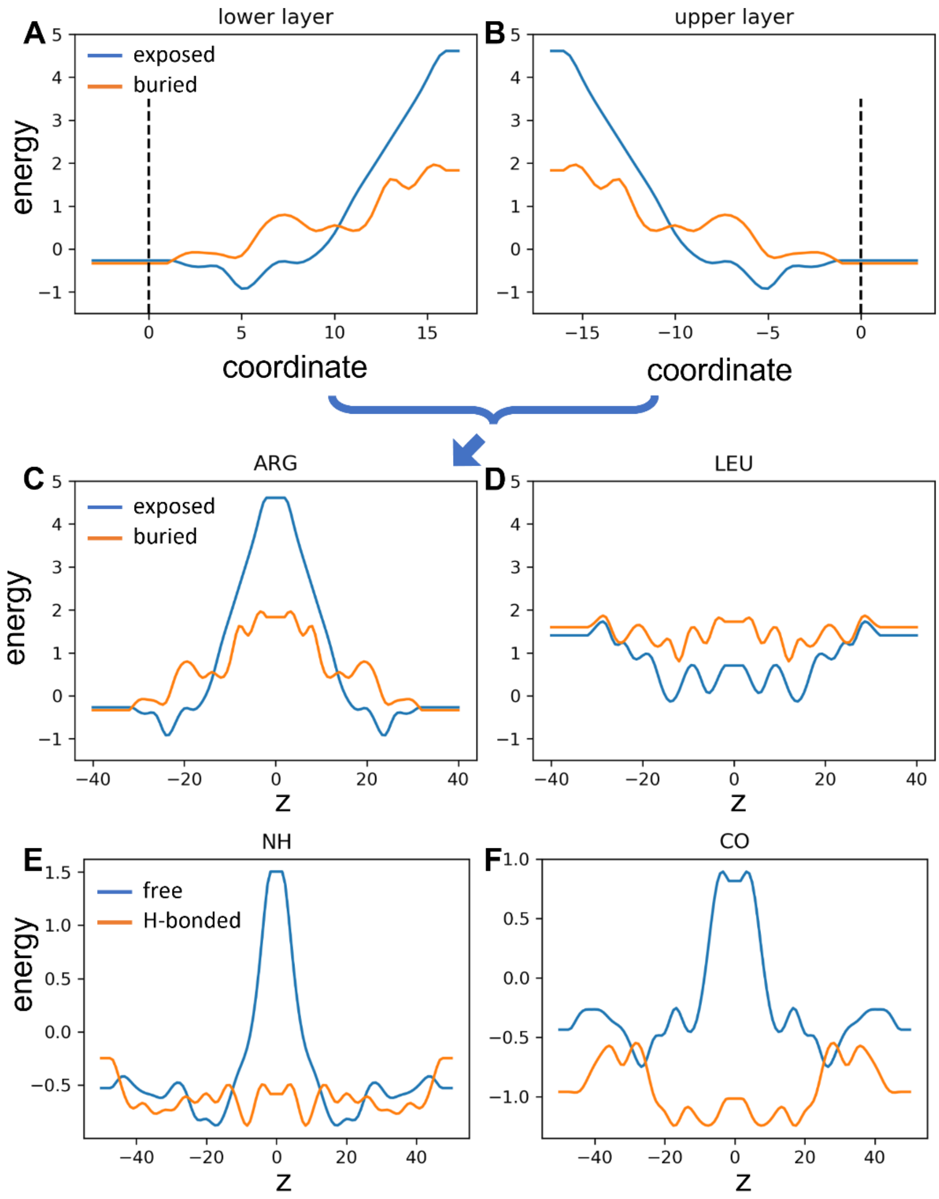

Figure S4. Examples of terms in the new protein-membrane burial potential. A. The Cβ-based potential contains components for lipid exposed and protein buried states, as well as upper and lower halves of the bilayer. However, the halves were forced symmetric during training. The coordinates are referenced to the boundaries of the bilayer. B. Residue-specific examples of the Cβ-based potential for a hydrophilic and a hydrophobic residue. The continuity between the upper and lower halves of the potential is depicted and now the referencing is from the center of the bilayer at Z=0. C. The unsatisfied H-bonding term for backbone NH and CO contains components for free acceptors and donors and a reference potential for when H-bonded.

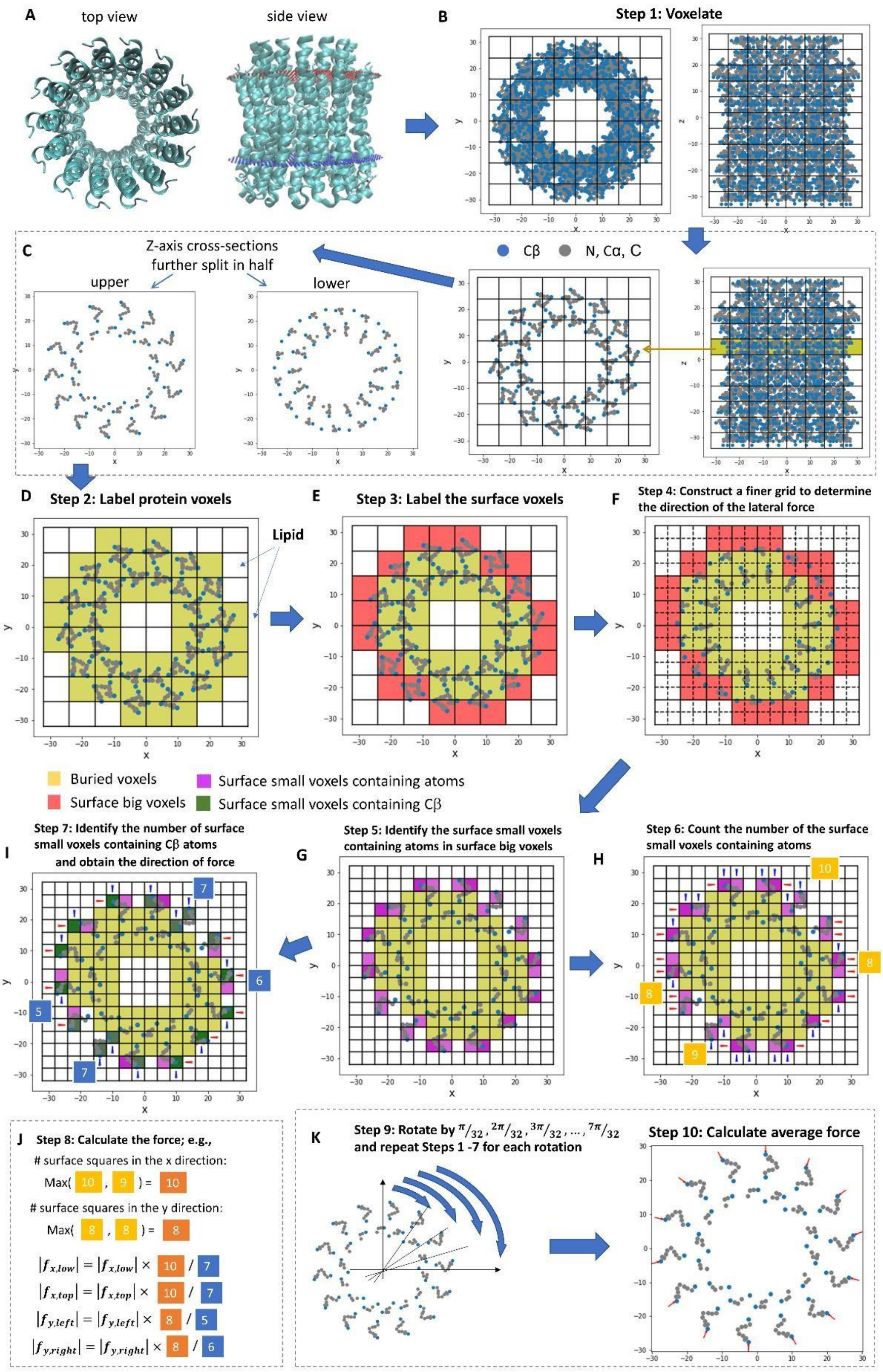

Figure S5. Steps in the Upside lateral pressure algorithm.

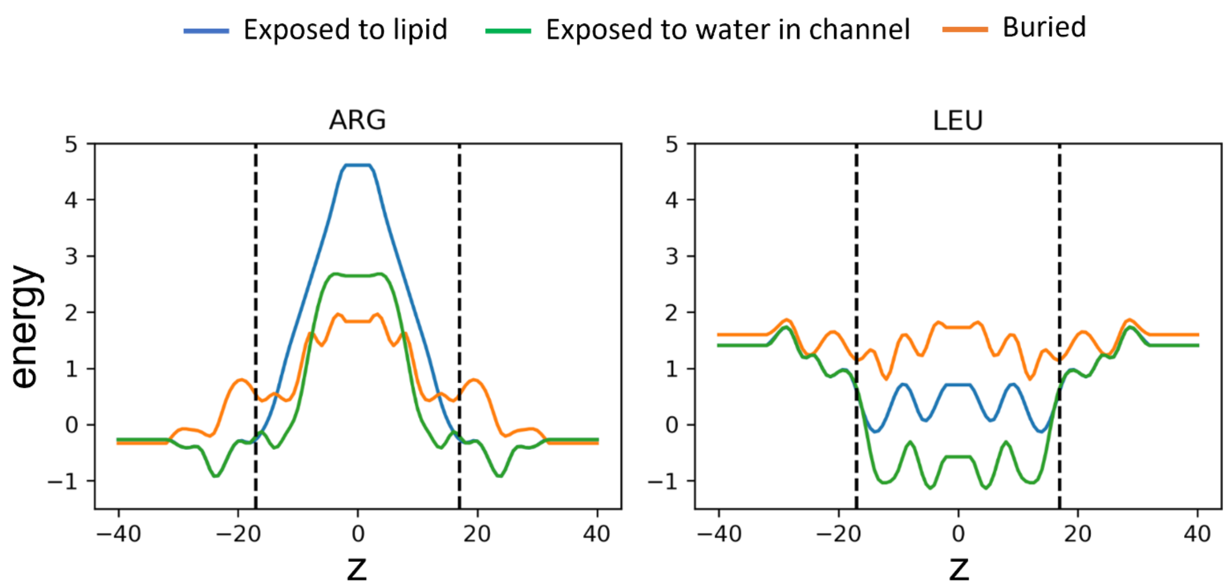

Figure S6. Protein-membrane burial potential components for membrane channel proteins. The lipid exposed and protein buried terms are the same as in Fig. S5, but now there is an additional term for residues exposed to water in the channel.
